# Doxorubicin-Conjugated Nanoparticles for Potential Use as Drug Delivery Systems

**DOI:** 10.3390/nano15020133

**Published:** 2025-01-17

**Authors:** Alua Imantay, Nariman Mashurov, Balnur A. Zhaisanbayeva, Ellina A. Mun

**Affiliations:** School of Sciences and Humanities, Nazarbayev University, Astana 010000, Kazakhstan; alua.imantay@alumni.nu.edu.kz (A.I.); nariman.mashurov@nu.edu.kz (N.M.); balnur.zhaisanbayeva@nu.edu.kz (B.A.Z.)

**Keywords:** drug delivery, doxorubicin, metallic nanoparticles, organosilica nanoparticles, polymeric nanoparticles

## Abstract

Doxorubicin (DOX) is one of the most widely used chemotherapy drugs in the treatment of both solid and liquid tumors in patients of all age groups. However, it is likely to produce several side effects that include doxorubicin cardiomyopathy. Nanoparticles (NPs) can offer targeted delivery and release of the drug, potentially increasing treatment efficiency and alleviating side effects. This makes them a viable vector for novel drug delivery systems. Currently, DOX is commonly conjugated to NPs by non-covalent conjugation–physical entrapping of the drug using electrostatic interactions, van der Waals forces, or hydrogen bonding. The reported downside of these methods is that they provide a low drug loading capacity and a higher drug leakage possibility. In comparison to this, the covalent conjugation of DOX via amide (typically formed by coupling carboxyl groups on DOX with amine groups on the nanoparticle or a linker, often facilitated by carbodiimide reagents), hydrazone (which results from the reaction between hydrazines and carbonyl groups, offering pH-sensitive cleavage for controlled release), or disulfide bonds (formed through the oxidation of thiol groups and cleavable by intracellular reducing agents such as glutathione) is more promising as it offers greater bonding strength. This review covers the covalent conjugation of DOX to three different types of NPs—metallic, silica/organosilica, and polymeric—including their corresponding release rates and mechanisms.

## 1. Introduction

**Figure 1:**
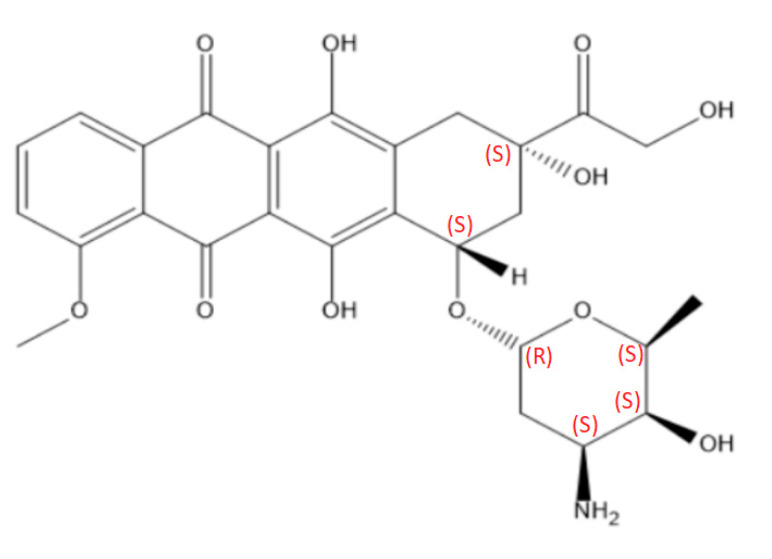
Doxorubicin structure.

Doxorubicin (DOX, Figure 1) is one of the most widely used drugs in the treatment of cancer in both adults and children. Initially, a compound called daunorubicin was derived from the *Streptomyces peucetius* bacterium [1]. It has been successfully used in patients with cancer for almost 20 years when the drug was found to cause fatal cardiac arrest [2]. Due to this serious side effect, daunorubicin was structurally modified to reduce its toxicity. The resulting compound was called doxorubicin. Currently, DOX is one of the first-line drugs in the treatment of breast, lung, bladder, thyroid, and bone tumors, as well as leukemias and sarcomas [3]. The downsides of doxorubicin therapy include fast excretion from the body and a low tumor-reaching rate, leading to the administration of higher doses. Consequently, this leads to the drug having a high cardiac toxicity level. For example, according to the protocol for the treatment of solid tumors, the maximum cumulative dose a patient can receive is 550 mg/m^2^ every three weeks [4]. At this dose, acute cardiac toxicity is recorded in 11% of patients. Cardiac toxicity then leads to the development of doxorubicin cardiomyopathy, which frequently results in heart failure. Other serious side effects of DOX include alopecia, vomiting, bone marrow suppression, and constant fatigue [5]. Due to the hydrophilicity of free doxorubicin, it can be readily absorbed in healthy tissues. As a result, the side effects are inevitable in intravenous administration.

**Figure 2:**
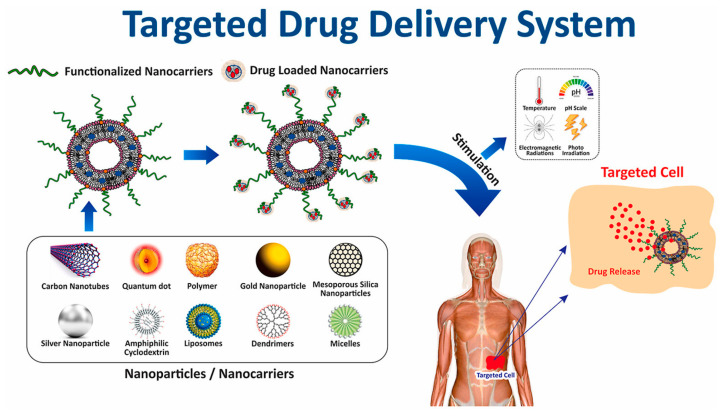
Types of targeted drug delivery systems [19]. Image adapted with permission from ref. [19]. Copyright 2021, Elsevier.

As such, there is a high need for targeted and controlled novel drug delivery systems that can alleviate the side effects of chemotherapy and increase treatment efficacy. Currently, several different carriers, such as quantum dots, carbon nanotubes, dendrimers, polymers, cyclodextrins, liposomes, micelles, and porous/non-porous nanoparticles (NPs), are available (Figure 2) [6]. Quantum dots are semiconductor nanocrystals that exhibit size-dependent optical properties, and in drug delivery systems, they are employed for targeted therapy and diagnostic imaging due to their tunable fluorescence and biocompatibility [7]. Carbon nanotubes are cylindrical structures with nanometer-scale diameters and axial symmetry, which confer distinct properties that can be utilized in the diagnosis and treatment of cancer [8]. Dendrimers are highly branched, monodisperse macromolecules with a well-defined, tree-like structure, and in drug delivery systems, they are utilized for their controlled drug release, high drug loading capacity, and ability to target specific cells or tissues through surface functionalization [9]. Polymers are large macromolecular compounds composed of repeating structural units, and in drug delivery systems, they are employed for their versatility in forming biodegradable carriers, enabling controlled drug release, enhancing drug stability, and facilitating targeted delivery through surface modification [10]. Cyclodextrins are cyclic oligosaccharides composed of glucose units, and in drug delivery systems, they are used to improve drug solubility, enhance stability, and control the release of hydrophobic drugs through encapsulation within their toroidal cavity [11]. Liposomes are lipid-based vesicles composed of one or more phospholipid bilayers, and in drug delivery systems, they are utilized for their ability to encapsulate both hydrophilic and hydrophobic drugs, enhance drug stability, reduce toxicity, and enable targeted delivery through surface modification [12]. Micelles are amphiphilic self-assembled nanoparticles formed from surfactant molecules, and in drug delivery systems, they are utilized for encapsulating hydrophobic drugs in their core, enhancing solubility, improving bioavailability, and enabling targeted drug release [13]. Nanoparticles are submicron-sized particles with a high surface-area-to-volume ratio, and in drug delivery systems, they are used to improve drug solubility, control release, and enable targeted delivery; they can be classified into porous nanoparticles, which allow for drug encapsulation within their internal structure, and non-porous nanoparticles, which primarily function through surface attachment or coating for sustained release [14,15]. The latter is of high interest due to several properties: good stability, low toxicity, easy synthesis routes, an ability to control their size, and, most importantly, an ability to functionalize their surface with different molecules, including chemotherapy drugs [16]. Among different nanoparticles, gold nanoparticles can be considered as the most feasible ones due to their biocompatibility, ease of surface functionalization, tunable size and shape, high drug loading capacity, controlled release ability, unique optical properties for imaging, low immunogenicity, and scalability, making them highly suitable for targeted and efficient therapeutic applications [17,18].

Doxorubicin can be conjugated to nanoparticles both covalently and non-covalently. Non-covalent conjugation involves the physical adsorption of the drug via electrostatic forces, van der Waals forces, or hydrogen bonding [19]. Although non-covalent conjugation has an easier synthesis procedure and requires fewer chemicals, it was reported to have a low drug loading capacity (DLC) and a higher probability of drug leakage during blood circulation. This is due to non-covalent linkages being weaker compared to their covalent counterparts [20]. The literature shows instances of 50% doxorubicin leaking prematurely in the case of electrostatic attachment. This can seriously harm healthy tissues before the drug reaches the target [21]. A different study showed that 5(6)-carboxyfluorescein conjugated to polymeric NPs was very rapid at around 30% in the first 2 min [12]. Such data show that conjugated drugs could be possibly released prematurely before reaching the target tissue. As such, this paper reviews the covalent conjugation of doxorubicin to three different types of nanoparticles—metallic, silica/organosilica, and polymeric—including their corresponding release mechanisms and rates.

## 2. Metallic Nanoparticles

Metallic nanoparticles are the most prevalent and the most studied type of doxorubicin delivery systems in the literature. The main reason for their popularity is their easy surface functionalization compared to other types of nanoparticles [22,23]. Typical metals used for the synthesis of nanoparticles include gold, silver, and iron.

Metallic nanoparticles, including gold, silver, iron, and copper nanoparticles, offer several advantages over other drug delivery carriers due to their unique physicochemical properties. These nanoparticles possess high surface-area-to-volume ratios, which allow for enhanced drug loading capacity and efficient release mechanisms. Additionally, their size and shape can be precisely tailored during synthesis, offering control over their behavior in biological systems. For example, MNPs like silver nanoparticles (AgNPs) exhibit antimicrobial properties, which can be beneficial for drug delivery applications targeting infections or chronic wounds [24,25]. Moreover, their surface can be easily functionalized with various ligands, such as antibodies or peptides, enabling targeted drug delivery and reducing off-target effects. The versatility of MNPs is also evident in their ability to be synthesized through sustainable methods, such as using agri-food waste extracts for green synthesis, which improves their biocompatibility and environmental safety [26]. Furthermore, metallic nanoparticles have shown effectiveness in food preservation applications, where they are incorporated into edible coatings to enhance the physicochemical and microbiological properties of seafood, demonstrating their broad utility and stability [27]. Overall, metallic nanoparticles stand out for their multifunctionality, ease of modification, and tunable properties, making them highly effective carriers in drug delivery systems.

**Figure 3:**
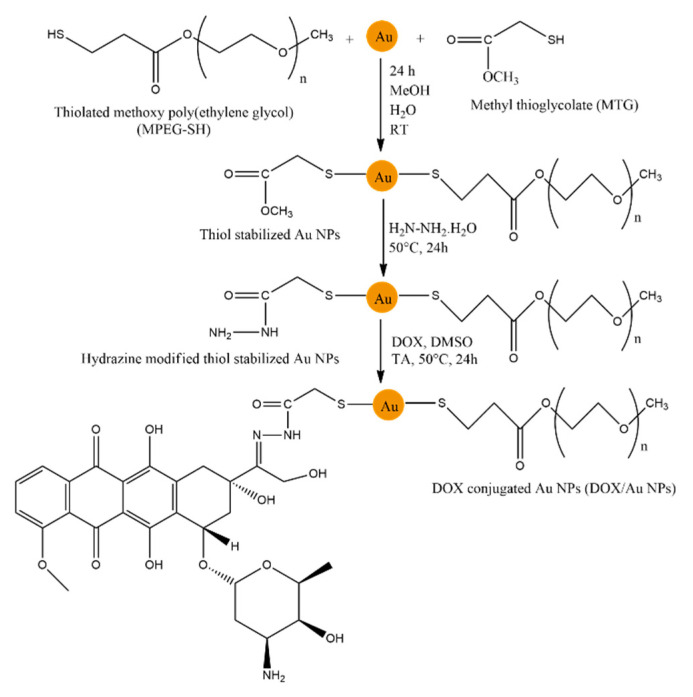
The synthesis mechanism of thiol-stabilized DOX-conjugated gold nanoparticles [31].

Among them, the covalent conjugation of doxorubicin to gold nanoparticles received significant attention. The successful covalent conjugation of DOX to gold nanoparticles was achieved via the formation of a hydrazone bond (Figure 3) [28]. First, the gold nanoparticles were stabilized using thiolated methoxy polyethylene glycol (MPEG-SH) and methyl thioglycolate (MTG). Since gold nanoparticles themselves have low solubility, PEG can increase this parameter due to its hydrophilic nature. After the stabilization of nanoparticles, the reaction with hydrazine was performed to provide sites for DOX conjugation. As a result, DOX was covalently conjugated via a hydrazone bond between MTG moiety and a drug. The average diameter of the nanoparticles was measured to be 6 nm using Transmission Electron Microscopy (TEM).

The implications of this work are significant for improving cancer therapy using gold nanoparticles (AuNPs) as drug delivery vehicles. The covalent conjugation of doxorubicin (DOX) to AuNPs via a hydrazone bond enables controlled drug release, particularly in the acidic tumor microenvironment, reducing systemic toxicity. The use of PEGylation to stabilize nanoparticles addresses their solubility issues, enhancing the circulation time and biocompatibility, as demonstrated in previous studies [29]. Furthermore, the small size of the nanoparticles (6 nm) facilitates efficient cellular uptake and tumor accumulation, leveraging the enhanced permeability and retention (EPR) effect, which has been widely observed in nanoparticle-based therapies [30].

**Figure 4:**
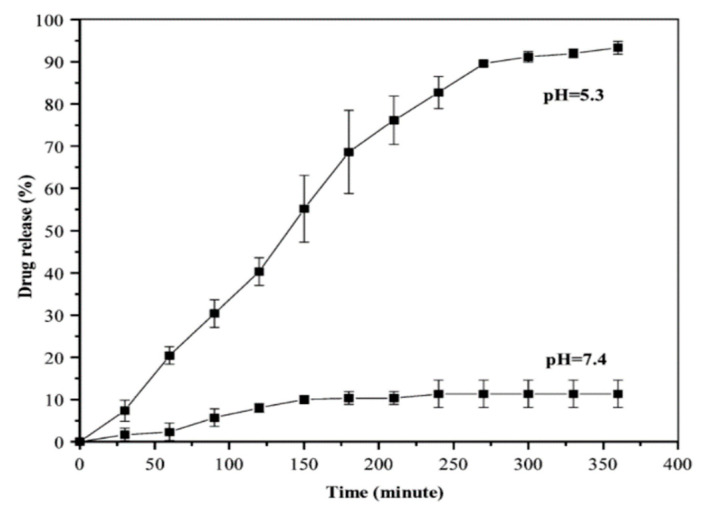
Percentage of DOX released at pH 5.3 and 7.4 [31]. Adapted with permission from ref. [31]. Copyright 2009, RSC.

Cancer cells tend to have a lower pH due to the accumulation of lactic acid [32]. Thus, after the internalization of the nanoparticle–drug conjugate via endocytosis, the drug can be released by the breakage of the hydrazone bond under the acidic environment of tumor cells. As such, the drug release rate was studied in vivo using two different pH environments: 5.3 and 7.4 (tumor and healthy cells, respectively). At the pH of healthy cells, only 10% of the drug was released, while at pH 5.3, 80% of the drug was released after a 5 h period (Figure 4). The results show that DOX will be successfully released from the nanoparticles after entering the cancer cell and will not be released under a physiological pH. The findings of this study have significant implications for targeted cancer therapy as they demonstrate the potential of pH-responsive drug delivery systems for the selective release of doxorubicin (DOX) within the acidic tumor microenvironment. The ability of the nanoparticle–drug conjugate to release DOX in response to the lower pH of cancer cells, while minimizing release in healthy tissues, can enhance therapeutic efficacy while reducing systemic toxicity [33]. This approach leverages the unique properties of tumor-associated acidosis, which has been widely observed in cancer cells [34], to facilitate more precise and controlled drug delivery, ultimately improving treatment outcomes and minimizing side effects.

**Figure 5:**
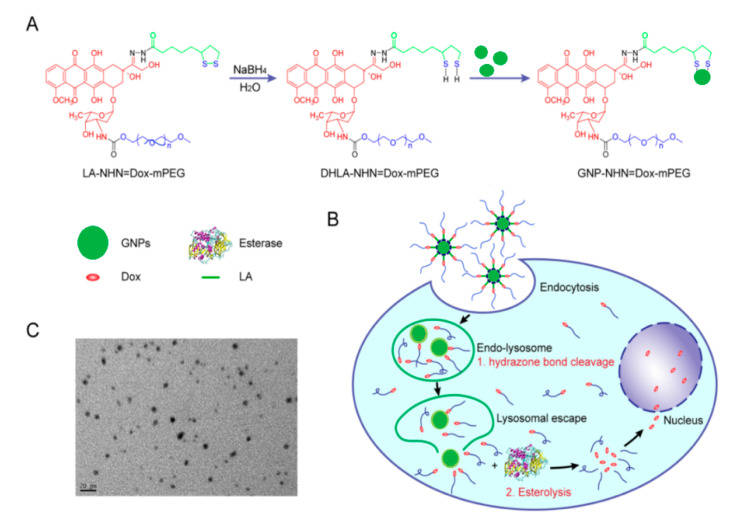
(**A**) Structure of DOX-conjugated gold nanoparticles. (**B**) Drug release mechanism in cancer cell environment. (**C**) TEM image of produced gold nanoparticles [35]. Adapted with permission from ref. [35]. Copyright 2017, American Chemical Society.

Doxorubicin itself can also be modified before conjugation to nanoparticles to improve the stability and solubility of the complex. DOX was modified with lipoic acid (LA) on the carbonyl group and polyethylene glycol (PEG) on the amino group [35]. The drug was covalently conjugated to lipoic acid via the formation of a hydrazone bond, resulting in the formation of LA-NHN = DOX-mPEG (Figure 5). The lipoic acid was then reduced to dihydro lipoate (DHLA) via a ring-opening reaction, which resulted in DHLA-NHN = DOX-mPEG containing thiol groups. Gold nanoparticles were then conjugated by disulfide bonding. The NP–drug conjugate was shown to exhibit a small size (179.0 ± 7.5 nm) that allows it to efficiently escape the reticuloendothelial system. The coating with PEG increased the solubility of the conjugate by 30 mg/mL compared to the solubility of doxorubicin hydrochloride (10 mg/mL). The drug loading capacity was determined to be 27.3%, which is higher than the values reported for non-covalent drug conjugation (10–15%).

**Figure 6:**
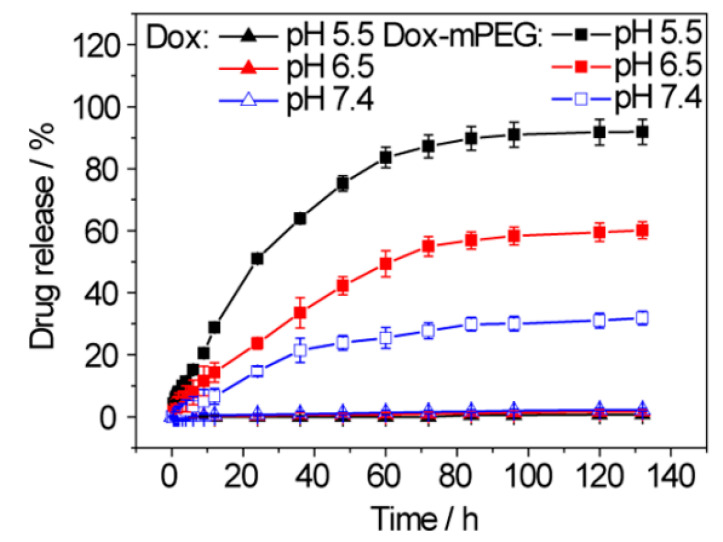
Percentage of DOX released at pH 5.5, 6.5, and 7.4 from free DOX and DOX-mPEG [35]. Adapted with permission from ref. [35]. Copyright 2017, American Chemical Society.

The drug release mechanism was based on the two-step bond breakage. First, the hydrazone bond is broken in the acidic lysosome environment, releasing the DOX-mPEG. Then, the high esterase concentration in tumor cells leads to the hydrolytic release of free DOX. At pH 5.5, 91.8% of DOX was released, while at pH 7.4, only 31.9% of the DOX was released (Figure 6). The three-fold difference between the release in tumor and healthy cells indicates that the conjugate will be stable during circulation and rapidly released after entering the cancer environment.

The implications of this research suggest that modifying doxorubicin (DOX) before conjugation with nanoparticles significantly enhances both the stability and solubility of the drug, leading to more effective delivery systems for cancer treatment. The modification of DOX with lipoic acid and polyethylene glycol (PEG) not only increases the drug’s solubility but also improves its loading capacity compared to traditional non-covalent conjugation methods [36]. The use of gold nanoparticles conjugated with disulfide bonds allows for better evasion of the reticuloendothelial system, which is crucial for enhancing the bioavailability of the drug in targeted cancer therapy [37]. Additionally, the two-step drug release mechanism, triggered by the acidic environment and esterase activity in cancer cells, enables controlled and selective drug release, further enhancing therapeutic efficacy and minimizing side effects in healthy tissues.

Magnetic iron oxide nanoparticles (MIONs) are another frequently used type of metallic nanoparticles due to their superparamagnetic properties [38]. Since they can be affected by magnetic fields, it is possible to guide the drug to the target. Moreover, MIONs are widely used to monitor real-time drug delivery using magnetic resonance imaging (MRI). However, due to their high surface energy, they tend to agglomerate rapidly [39]. In addition to this, they demonstrate high reactivity that usually results in a loss of magnetism [40]. Coating nanoparticles with biomolecules is one of the possible solutions to the above-mentioned problems associated with MIONs. As such, the covalent conjugation of doxorubicin to metallic nanoparticles can be an excellent drug delivery system and can increase stability by lowering the oxidation rate of naked nanoparticles.

**Figure 7:**
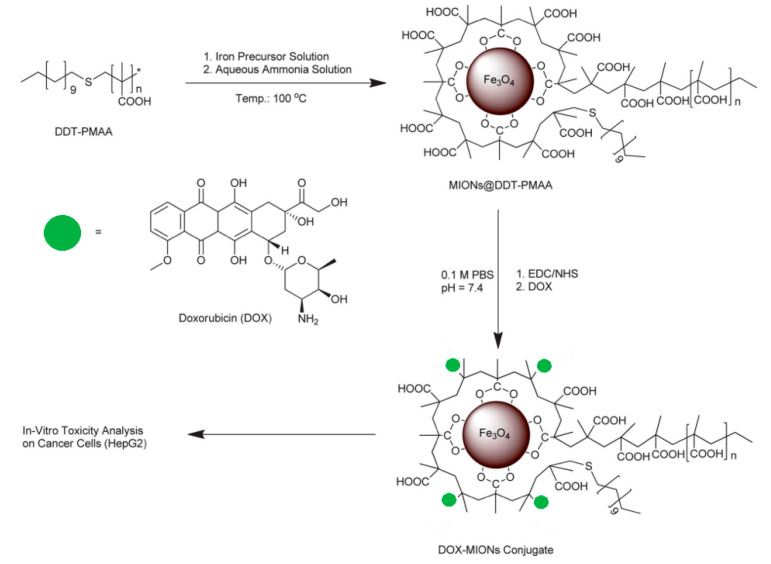
Synthesis of DOX-MION conjugate [43]. DOX release mechanism. Adapted with permission from ref. [43]. Copyright 2013, Royal Society of Chemistry.

The formation of MIONs via a single-step synthesis method was reported by using thioether end-functionalized polymer ligand dodecanethiol–polymethacrylic acid (DDT–PMAA) [20]. It was then reacted with an iron precursor that allowed for the formation of ultra-small (4.6 ± 0.7 nm) magnetic iron oxide nanoparticles exhibiting a high number of -COOH groups at their surface (Figure 7). DOX was then covalently conjugated via the formation of an amide bond between those carboxyl groups and amino groups of the drug. The resulting DOX-coated MIONs showed good stability compared to the naked MIONs. The drug conjugation efficiency was determined to be 60%; this is higher compared to that of MIONs non-covalently/electrostatically bound to magnetic iron oxide nanoparticles. This observation proves that covalent conjugation can allocate more drug capacity due to the stronger nature of covalent bonds. The implications of this study highlight the potential for improving the therapeutic efficacy of cancer treatment by enhancing the stability and drug loading capacity of nanoparticle-based drug delivery systems. The use of covalent conjugation, as demonstrated by the high drug conjugation efficiency of 60%, offers a significant advantage over traditional non-covalent binding methods, which typically result in lower drug loading capacities. This finding suggests that the covalent attachment of drugs like doxorubicin to magnetic iron oxide nanoparticles (MIONs) could not only enhance drug stability during circulation but also provide more efficient and sustained drug release at targeted tumor sites. Additionally, the method of using thiol-functionalized polymer ligands to stabilize nanoparticles opens new avenues for designing versatile and customizable drug delivery systems with the potential to minimize off-target effects and improve the overall therapeutic index. Such advancements could lead to more effective cancer therapies with fewer side effects, offering significant improvements over existing drug delivery technologies [41,42].

Despite all the benefits provided by the conjugated complexes of metallic nanoparticles for DOX delivery, there are a few drawbacks of the proposed system, such as cost-effectiveness, toxicity response to inorganic components, and longevity of elimination from the body [28].

## 3. Silica/Organosilica Nanoparticles

Silica and organosilica nanoparticles are considered to be relatively novel drug delivery systems. They tend to have excellent stability and low toxicity. As opposed to inorganic NPs, organosilica NPs contain an organic component that could potentially positively affect their degradation and toxicity profiles [44]. The conjugation of doxorubicin to silica/organosilica NPs mainly happens via physical entrapping in the pores [45]. However, since the electrostatic forces are weak, there is high drug leakage. Covalent conjugation was used as an alternative since it is significantly stronger. As a result, premature drug release could be avoided. This type of conjugation arose only recently; therefore, the literature on doxorubicin covalent conjugation to organosilica/silica NPs is limited. 

**Figure 8:**
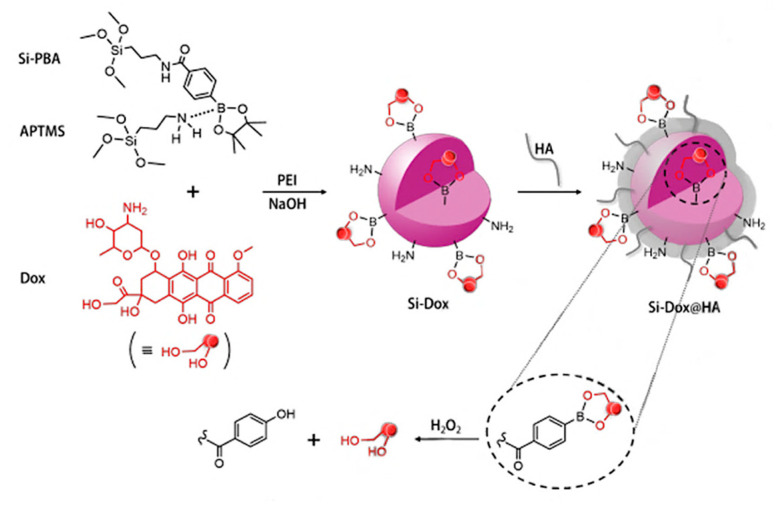
Synthesis of DOX-conjugated organosilica nanoparticles using PBA and APTMS [46]. Adapted with permission from ref. [46]. Copyright 2019, WILEY.

DOX was covalently bound to organosilica nanoparticles via phenyl borate ester bonds (Figure 8) [46]. Phenylboronic acid (PBA) coupled with 3-aminopropyltriethoxysilane (APTMS) was used as a precursor for the synthesis of nanoparticles. DOX was conjugated to a phenylboronic moiety of NPs. However, the obtained conjugate was too large, allowing the immune system to easily detect and excrete it from the body. As such, polyethyleneimine (PEI) was introduced to the synthesis process to allow for size control.

Several different concentrations of the reagents were tested to determine the most suitable reaction conditions (Table 1).

**Figure 9:**
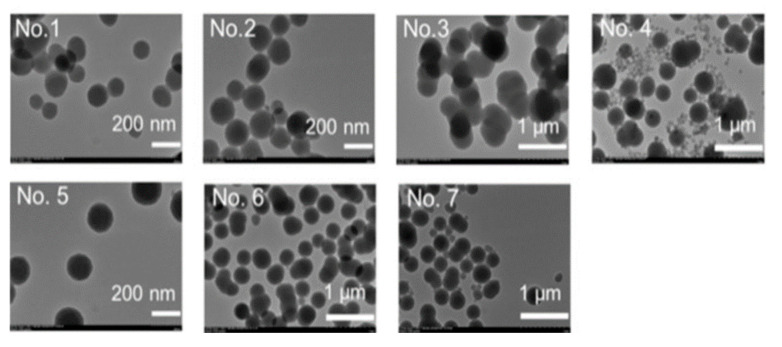
TEM images of synthesized nanoparticles [46]. Adapted with permission from ref. [46]. Copyright 2019, WILEY.

A high concentration of APTMS resulted in the production of nanoparticles with large diameters, while a high concentration of PEI led to the synthesis of very dispersed NPs (Figure 9). However, it also resulted in a low loading capacity. Still, the second condition was chosen as the optimal reaction condition. After successful conjugation, the DOX-NP conjugate was coated with hyaluronic acid (HA) to allow for better targeting since HA has a high affinity to CD-44 glycoproteins that are usually overexpressed in cancer cells.

**Figure 10:**
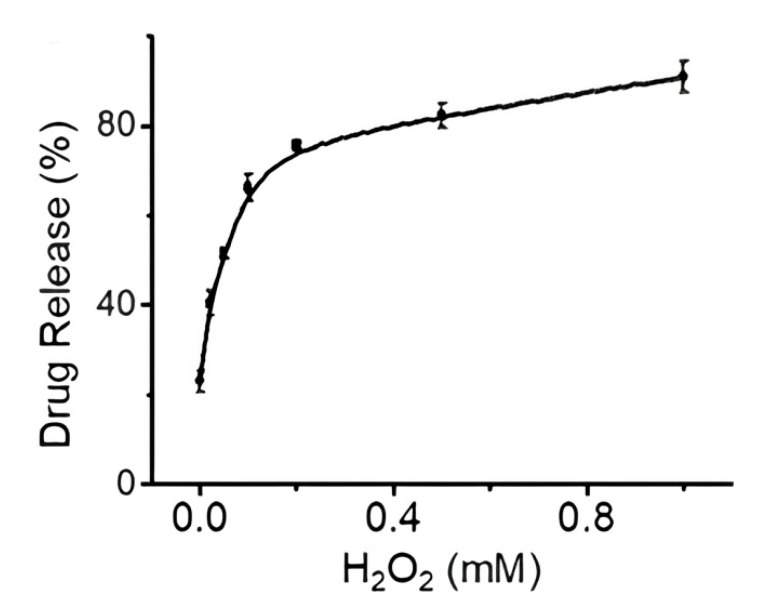
Percentage of DOX released at different H_2_O_2_ concentrations [46]. Adapted with permission from ref. [46]. Copyright 2019, WILEY.

Drug release was studied in an H_2_O_2_-containing medium since cancer cells tend to have higher concentrations of hydrogen peroxide (up to 1 mM) compared to healthy cells. The phenyl borate ester bond between doxorubicin and NP can be easily hydrolyzed by H_2_O_2_, resulting in successful drug release in the tumor environment. The release rate at 100 μM H_2_O_2_ and pH 6.5 (a cancer environment tends to be more acidic) was equal to 80% (Figure 10).

This study presents the development of doxorubicin (DOX)-conjugated organosilica nanoparticles (NPs) as a targeted drug delivery system with potential therapeutic advantages in cancer treatment. The incorporation of polyethyleneimine (PEI) into the nanoparticle synthesis process allowed for precise control over the particle size, addressing the challenge of immune system recognition and clearance commonly associated with large nanoparticles [47]. Additionally, coating the nanoparticles with hyaluronic acid (HA) facilitated the specific targeting of CD-44-overexpressing cancer cells, improving delivery efficiency to the tumor site. The phenyl borate ester bond linking DOX to the NPs was designed to undergo hydrolysis in the presence of hydrogen peroxide, a hallmark of the tumor microenvironment, ensuring controlled drug release in acidic conditions [48]. This study underscores the significance of the nanoparticle size, surface modification, and environmental responsiveness in enhancing the specificity, efficacy, and safety of nanoparticle-based drug delivery systems for cancer therapy.

**Figure 11:**
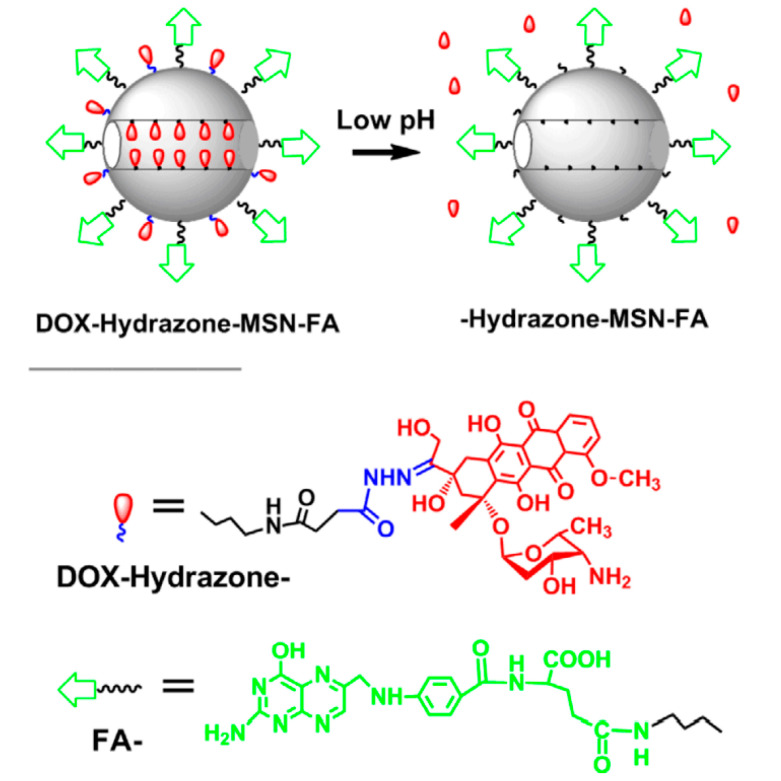
Structure of DOX-Hyd-MSN-FA [49]. Adapted with permission from ref. [49]. Copyright 2011, IOP Science.

Mesoporous silica nanoparticles (MSNs) can also be used for the covalent conjugation of doxorubicin [49]. DOX was conjugated to MSNs via the formation of hydrazone bonds (Figure 11). The conjugate was then coated with folic acid (FA) to target folate receptors that are typically overexpressed in tumor cells. The conjugate was found to exhibit a small diameter of 180 nm. It is crucial to keep the size smaller than 300 nm since larger particles can easily be detected by the immune system, leading to their faster clearance.

**Figure 12:**
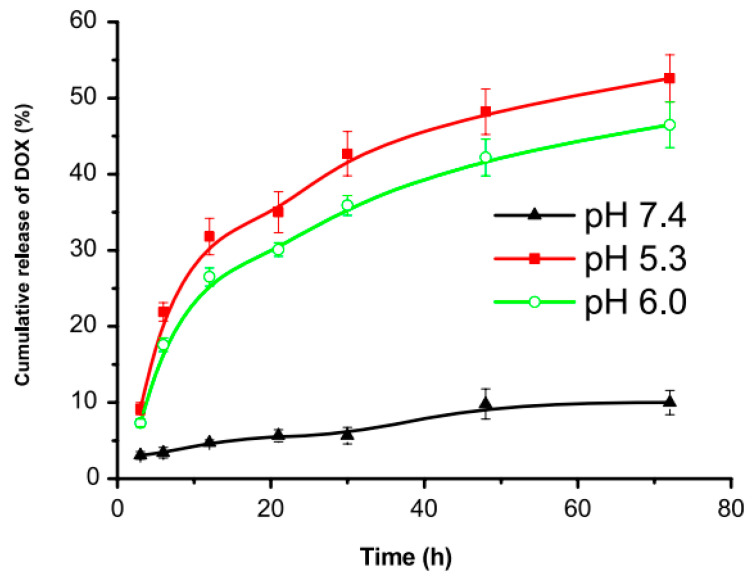
Cumulative release rate of DOX from DOX-MSN conjugate at pH 5.3, 6.0, and 7.4 [49]. Adapted with permission from ref. [49]. Copyright 2011, IOP Science.

The DOX-MSN conjugate can enter the cell via endocytosis, where the hydrazone bone can be easily cleaved by the acidic environment. As such, the drug release rate was studied at different pH media: 5.3, 6.0, and 7.4. The results after 24 h (Figure 12) show 40%, 30%, and 5% release rates, respectively. The slow release of DOX at a physiological pH indicated the higher stability of the nanoparticles during circulation, which can significantly decrease the side effects.

## 4. Polymeric Nanoparticles

Polymeric nanoparticles are another candidate for drug delivery systems used for the covalent conjugation of doxorubicin. They provide good stability and easy conjugation [50].

The idea of covalent polymer–drug conjugates, where drugs are attached to a polymer backbone via labile bonds, was introduced by Helmut Ringsdorf. His model features three key components: a hydrophilic segment for solubility and non-toxicity, a drug-linked region, and a transport system to deliver the drug to target sites. These components are organized through methods like block or statistical copolymerization [51].

One of the first drug-conjugated polymeric nanoparticles was reported by Kopeček et al., who described an N-(2-hydroxypropyl)methacrylamide (HPMA) copolymer covalently attached to a doxorubicin molecule. The conjugate was designed to be cleaved via hydrolysis by specific enzymes in lysosomes [52] that had a reactive species in the monomers that could link the drug. This was further developed by Peng et al., where an HPMA copolymer was utilized as a drug carrier for prostate cancer treatment. Doxorubicin (DOX) was conjugated to the HPMA copolymer through a lysosomal cleavable linker and tumor-targeting and -penetrating peptide iRGD, which is selectively cleaved by MMP-2, an enzyme overexpressed in the prostate cancer microenvironment and closely linked to tumor progression [53]. The resulting formulation was effective in causing cell cycle arrest and cell death in both monolayer cell lines and spheroid cultures [53]. HPMA–doxorubicin conjugates were also shown to exhibit improved efficacy and to decrease toxicity, demonstrating a circulating half-life that was 15 times longer compared to that of the free drug [54]. The same conjugates were proposed for the targeted chemotherapy of primary and metastatic liver cancer, showing that the heart level of free DOX was reduced by 100-fold in 15 min [55]. The improved performance provided by the HPMA-DOX conjugates is attributed to the stealth effect of nanocarriers characterized by steric stabilization in retarding reticuloendothelial system clearance and prolonging blood circulation, resulting in enhanced pharmacokinetic profiles. This effect was mainly described for liposomes and polymeric micelles/nanoparticles [56].

**Figure 13:**
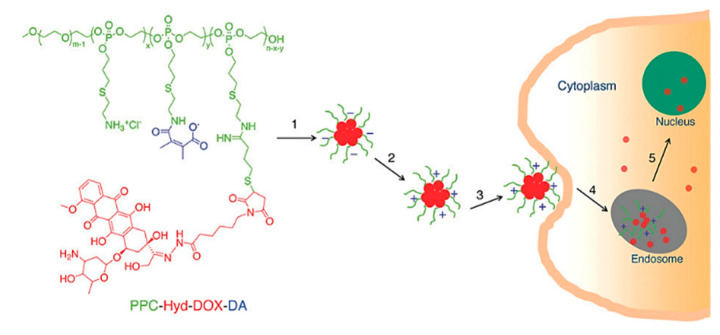
Structure and internalization route of PPC-Hyd-DOX-DA [57]. Adapted with permission from ref. [57]. Copyright 2011, American Chemical Society.

Polymeric nanoparticles were synthesized from cysteamine-modified polyallyl ethylene phosphate (PPC) [57]. Doxorubicin was then conjugated via the formation of a pH-responsive hydrazone bond. Free amino groups were modified with 3-dimethylmaleic acid (DA) to obtain a charge-conversional conjugate (Figure 13). The resulting nanoparticles, PPC-Hyd-DOX-DA, self-assembled in the water due to the hydrophobic nature of the DOX moiety. As a result, drug–NP conjugates with a negative surface charge were formed. After entering the extracellular tumor environment, the amide bonds were broken, and the conjugate acquired a positive charge that allowed for endocytosis through the negatively charged cell membrane.

**Figure 14:**
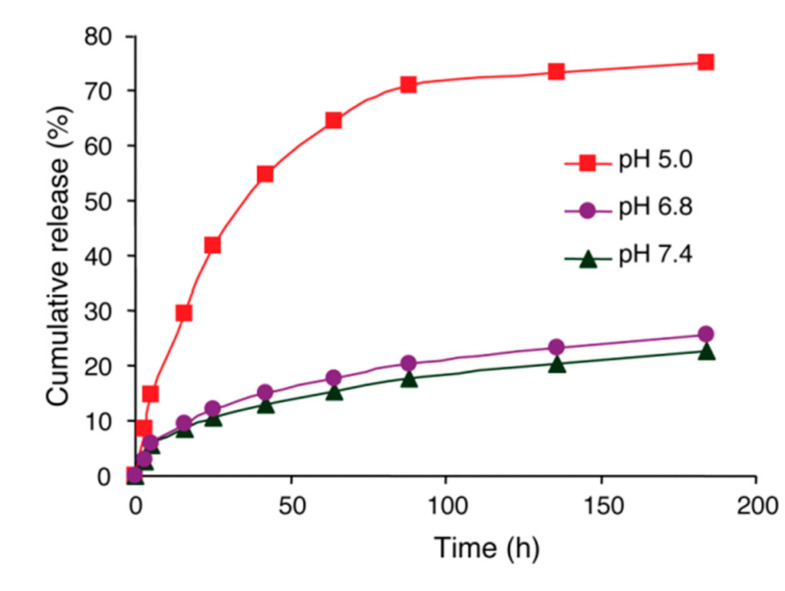
Cumulative release percentage of DOX from polymer–DOX NPs at pH 5.0, 6.8, and 7.4 [57]. Adapted with permission from ref. [57]. Copyright 2011, American Chemical Society.

In vivo drug release was studied using two media with different pH levels, (5.0 and 7.4), cancer and healthy cells, respectively. At pH 7.4, only 22.6% of DOX was released, whereas at pH 5.0, the value reached approximately 75% (Figure 14). Thus, the drug release profile shows that the hydrazone bonds attaching the drug to the nanoparticle are expected to be broken at the endosomal acidic pH of cancer cells. Moreover, it also shows that the conjugate is likely to be relatively stable during circulation before reaching the target cancer cells.

These findings indicate that pH-responsive nanoparticles, with charge-conversional properties, may offer a promising approach to enhance the specificity, stability, and therapeutic efficacy of drug delivery systems in cancer treatment [58]. This strategy can potentially minimize systemic toxicity and increase the efficiency of chemotherapy by ensuring that the drug is released specifically at the tumor site.

**Figure 15:**
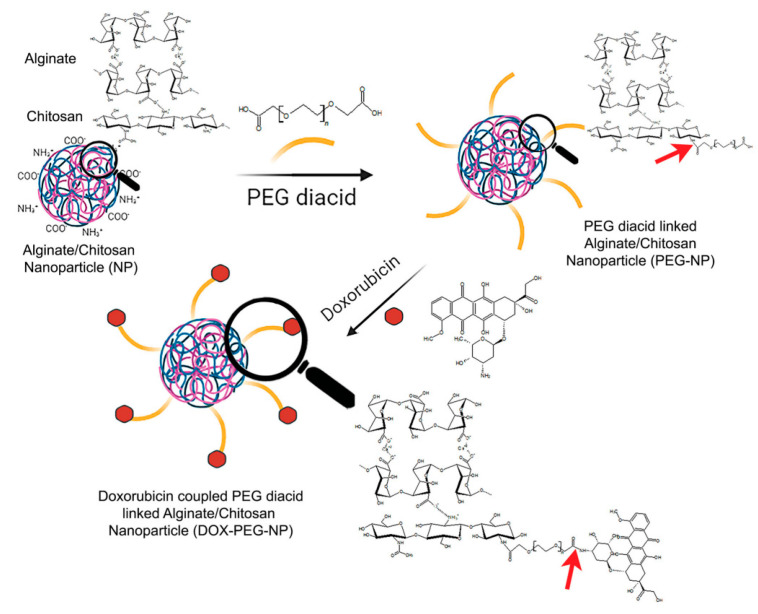
Modification with PEG and conjugation with DOX of alginate/chitosan NPs [59]. The arrows indicate the amide linkages between NPs and PEG, and between PEG and DOX. Adapted with permission from ref. [59]. Copyright 2021, Taylor & Francis.

Polymeric nanoparticles can also be synthesized by conjugating alginate and chitosan, which can be used in lung cancer treatment [59]. The main role of chitosan is to stabilize the polymer and allow the synthesis of more dispersed and small nanoparticles. The size distribution was assessed using Dynamic Light Scattering, and the average diameter was in the range of 60–65 nm, with PDI values of lower than 0.5. Particles of small size can easily enter tumor cells via an enhanced permeation and retention effect (EPR). To remove the steric hindrance of resulting nanoparticles, they were modified with PEG (Figure 15). Finally, doxorubicin was conjugated via the formation of an amide bond between polyethylene glycol and the drug. The nanoparticles showed excellent drug conjugation efficiency of 49.1 ± 3.1%.

**Figure 16:**
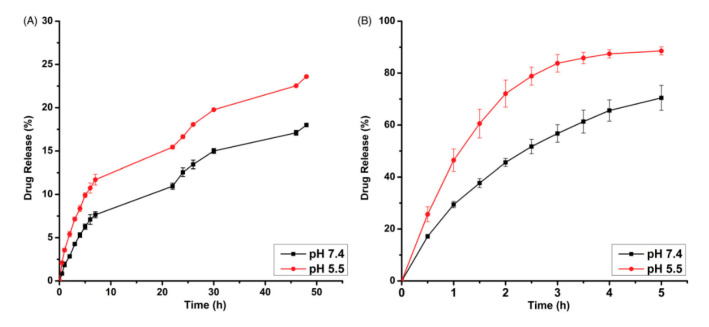
(**A**) Percentage of DOX released by DOX-NPs in pH 5.5 and 7.4. (**B**) Percentage of DOX released by free DOX in pH 5.5 and 7.4 [59]. Adapted with permission from ref. [59]. Copyright 2021, Taylor & Francis.

The DOX-NP drug release rate was studied at two different pH levels, 5.5 and 7.4. The results were also compared with the release rate of free doxorubicin (Figure 16). It was shown that 70–85% of free doxorubicin was released in both healthy and cancer cells. These findings clearly indicate that doxorubicin cannot differentiate between healthy and cancerous cells. The high release rate in healthy cells results in serious side effects associated with chemotherapy. DOX release from polymeric nanoparticles takes place via the breakage of an amide bond. At pH 5.5, the release rate reached 23.6%. In comparison, at a physiological pH of 7.4, the maximum release rate was 18%. The release rate is significantly lower compared to the release of free DOX, which indicates that polymeric nanoparticles can be used as drug delivery systems that can potentially decrease the negative side effects on healthy cells.

Despite the mentioned advantages of polymeric nanoparticles in drug delivery for cancer treatment, some serious drawbacks can limit their applicability. Difficulty in controlling their morphology is one of these; it greatly hinders the reproducibility of the results [60].

## 5. Improving Drug Release

**Figure 17:**
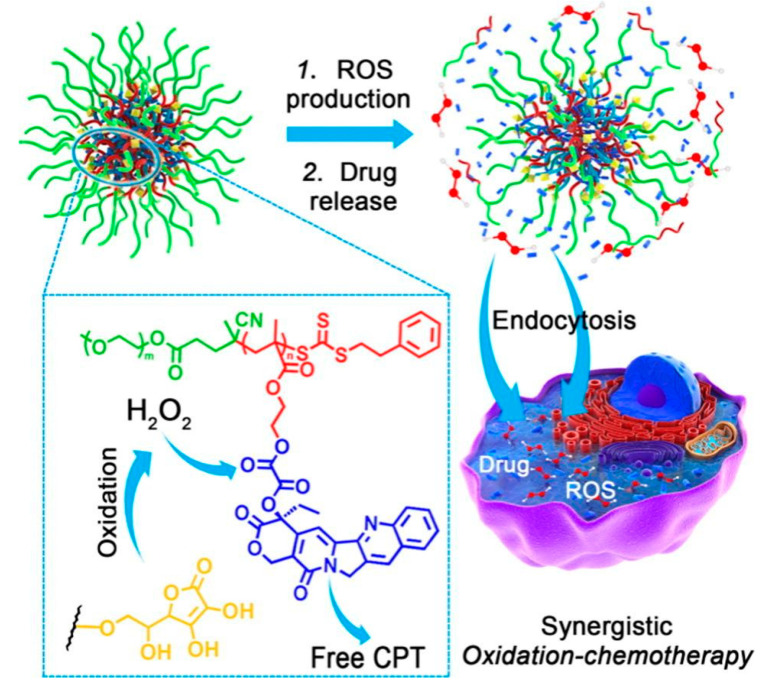
Schematic representation of H_2_O_2_-responsive PEG-based nanocarrier action [62]. Adapted with permission from ref. [62]. Copyright 2016, Elsevier.

As mentioned before, drug release efficacy remains a major concern in modern anti-cancer therapy. Pre-existing tumor microenvironments (TMEs) that include lower pH, hypoxia, and elevated numbers of immunosuppressing cells are one of the obstacles that decrease the efficacy of modern therapies [61]. Recently, new methods were proposed to reprogram the TME, increasing the efficacy of stimuli-responsive nanocarriers. One of the proposed methods comprises self-sustaining H_2_O_2_-responsive PEG-based nanocarriers (Figure 17) [62]. By incorporating prooxidants such as palmitoyl ascorbate, these delivery systems amplify the production of reactive oxygen species. There are two major ways in which it improves the efficiency of anti-tumor therapies: First, it exposes cancer tissue to higher levels of oxidative stress, promoting apoptotic cell death as a result. In addition to this, it can be used as a trigger for the release of chemotherapeutic agents (such as camphothecin (CPT)) from the nanocarriers.

**Figure 18:**
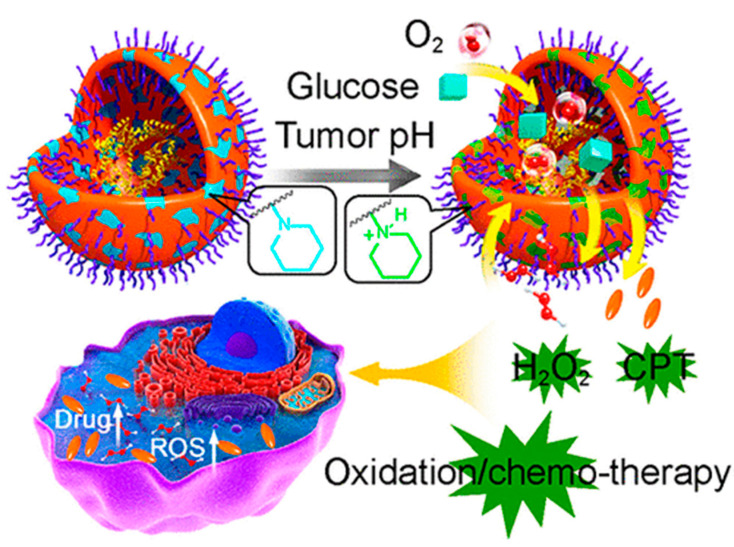
Schematic representation of glucose oxidase-containing polymersome nanocarrier action [63]. Adapted with permission from ref. [63]. Copyright 2017, American Chemistry Society.

In addition to this, tumor microenvironment-responsive nanoreactors were proposed [63]. These polymer prodrug-based nanoreactors were designed to specifically respond to acidic TMEs (Figure 18). Li et al. utilized polymersomes that contained glucose oxidase that were engineered to remain inert in normal tissues but became active upon exposure to the acidic pH of the tumor. Upon activation, nanoreactors produced H_2_O_2_, increasing oxidative stress. Simultaneously, H_2_O_2_ would cleave the chemical bonds in the prodrug, releasing an active chemotherapeutic agent, camphothecin.

These findings imply a considerable improvement in therapy efficiency as well as a decrease in off-target effects. Potentially, they can be used to develop novel doxorubicin delivery systems.

## 6. Clinical Development of DOX Conjugates

As was mentioned before, doxorubicin is a potent chemotherapeutic agent. Yet, it poses several debilitating side effects. Thus, there were numerous attempts to introduce its conjugates for clinical applications that would possibly improve its therapeutic potential (Table 2). In 1987, the clinical trial of DOX-containing liposomal formulation (OLV-DOX) was conducted [64]. However, the results show that there were several downsides of this doxorubicin delivery: the drug was released in plasma too quickly [64], causing unwanted cardiotoxicity as a result. In addition to this, the reticuloendothelial system was rapidly clearing the administered liposomes [65]. Thus, the clinical trial failed.

The first successful conjugate, Doxil^®^, was produced using the OLV-DOX framework. However, PEGylation was used this time to improve the circulation time. It was recommended for FDA approval in November 1995 [67,68]. A year later, it was commercially available in the USA as Doxil^®^ and in the EU under the brand name Caelyx^®^. The formulation consists of a PEGylated liposomal bilayer with a size of 80–90 nm, loaded with Dox [68]. It contains hydrogenated soy phosphatidylcholine, cholesterol, and methyl-distearoyl phosphor-ethanilamine PEG 2000 sodium salt in a weight ratio of 3:1:1 [68]. However, prolonged plasma circulation offered by PEGylation resulted in the appearance of palmar-plantar erythrodysesthesia (hand-foot syndrome) as a side effect [66].

In 2000, Myocet^®^ was approved by EMA in the EU and Canada. It is a non-PEGylated liposomal alternative to Doxil^®^/Caelyx^®^. The formulation includes liposomal membrane phosphatidylcholine and cholesterol, with doxorubicin physically entrapped within. The total size is 190 nm. It is primarily used for metastatic breast cancer (MBC) [70], providing similar efficacy to free DOX but with a significantly lower risk of cardiotoxicity and without hand-foot syndrome [69].

In 2002, the Department of Health approved the use of Lipo-Dox^®^ in Taiwan. It is a PEGylated liposomal formulation that contains 1,2-distearoyl-sn-glycero-3-phosphocholine (DSPC) [72]. This preparation was more stable as it showed less drug leakage, owing to DSPC’s saturated fatty acids [71]. It is used for MBC, ovarian cancer, and AIDS-related Kaposi’s sarcoma but offers limited therapeutic improvement over Doxil^®^ [66].

Another formulation, ThermoDox^®^, incorporated lyso-thermosensitive liposomes. These liposomes (around 100 nm) release doxorubicin in areas of mild hyperthermia (>40 °C) [75]. This, combined with radiofrequency ablation (RFA), was expected to target cancerous tissue, releasing doxorubicin locally. However, in 2020, during Phase III of clinical trials, namely the OPTIMA study, there was no significant therapeutic effect of ThermoDox^®^ + RFA treatment compared to RFA alone [73]. For now, the ThermoDox^®^ trial was halted, but the results yielded with this unique formulation show that it can be used for future thermosensitive drug delivery.

In 2011, a patent was granted to Livatag^®^, a formulation that utilized polymeric NPs formed with polyisohexylcyanoacrylate (100–300 nm) [3]. It was used for the treatment of advanced hepatocellular carcinoma, particularly in cases where first-line treatments like sorafenib failed. However, in 2017, during Phase III of the clinical trial, it was proclaimed a failure as it did not produce any better results compared to existing therapies [3]. Still, the patent was extended until 2031, indicating that new developments can be made for this formulation.

Polymer–drug conjugates have also been explored for DOX delivery in clinical studies. FCE28068/PK1 is an untargeted polymer conjugate using N-(2-hydroxypropyl)methacrylamide (HPMA) with a cleavable peptidyl linker [66]. Phase II trials, completed in 2009, demonstrated its efficacy in breast, colorectal, and non-small cell lung cancers with an extended plasma half-life and reduced cardiotoxicity [66]. FCE28069/PK2 builds upon PK1 by adding galactosamine for the active targeting of liver cancer through asialoglycoprotein receptors [66]. This targeted formulation remains in Phase II trials.

Polymeric micelles also represent a promising vector for DOX delivery. SP1049C, a micellar formulation using Pluronics^®^ L61 and F127, entered Phase III trials in 2007 for adenocarcinoma of the esophagus and gastroesophageal junction [66]. It has shown favorable toxicity profiles and promising therapeutic responses in clinical trials. NK911, another micellar formulation, uses poly-aspartic acid and polyethylene glycol to encapsulate DOX. Initially entering Phase I trials in 2001 and progressing to Phase II trials in 2004, NK911 demonstrated extended plasma retention and reduced off-target toxicity, particularly for metastatic pancreatic cancer [66].

More recently, novel formulations MM-302 and 2B3-101 were developed for the treatment of HER2-positive breast cancer [3] and breast cancer metastases in the brain, respectively [74]. The former one contains PEG-modified liposomes with doxorubicin and HER2-specific antibodies [74]. The latter one uses liposomal doxorubicin hydrochloride with glutathione ligands. Currently, both are in Phase II [74].

Overall, liposomes are the main drug delivery method used for doxorubicin delivery in the clinical setting, with more formulations being tested in different trial phases.

## 7. Conclusions

Doxorubicin treatment often leads to severe late-stage cardiotoxicity and damage to other vital organs, which can be life-threatening. Its toxicity is primarily linked to ROS and iron accumulation, though it also triggers immune system activation, alterations in gene expression, and disruptions in cardiac repair processes [76].

Moreover, doxorubicin is highly susceptible to multidrug resistance due to its mechanism of cellular entry via passive diffusion, making it vulnerable to protective mechanisms such as P-gp-mediated drug efflux. In contrast, most nanoparticles are internalized into cells through endocytosis, where the drug is cleaved from the nanoparticle in the acidic environment of endosomes. This process allows doxorubicin to be released directly into the cytoplasm, thereby bypassing the multidrug resistance effect (Gu et al., 2012 [77]).

Most reported nanocarriers of doxorubicin rely on noncovalent bonding, such as hydrophobic, electrostatic, and van der Waals forces. However, these interactions often lead to rapid drug release after administration, making it challenging to achieve controlled release. To address this issue, various strategies have been proposed to improve drug release kinetics, including the covalent attachment of the drug (Yang, Chen and Hu, 2014 [78]).

The covalent conjugation of doxorubicin to nanoparticles is a more efficient design of a drug delivery system than non-covalent conjugation. The latter cannot target delivery, while covalent conjugation results in the breakage of the bond between DOX and the nanoparticle in response to different environmental stimuli, such as the pH and hydrogen peroxide concentrations of tumor cells.

Moreover, covalent conjugation shows a significantly higher drug release percentage in a tumor environment compared to a healthy one. It proves that the drug will be preferentially released in the tissues affected by cancer due to environmental factors. Despite non-covalent conjugation being more prevalent due to its simplicity, covalent conjugation seems to be a more promising method in drug delivery due to its advantages.

The advanced properties of doxorubicin-conjugated nanoparticles, achieved through covalent bonding, create new opportunities in drug delivery systems. These nanoparticles enhance drug stability, minimize premature release at untargeted sites, and reduce systemic toxicity compared to conventional formulations or encapsulation techniques. Future advancements in personalized medicine will enable the incorporation of various targeting molecules onto the surface of doxorubicin-conjugated nanoparticles, further improving the precision and efficiency of targeted drug delivery. Moreover, we believe that a paradigm shift is occurring in the field of nanomedicine, where the advanced properties of inorganic nanoparticles will not only enhance drug delivery but also enable diverse applications in detection and controlled drug release, regulated by stimuli present within the cell.

## Figures and Tables

**Table 1:** Different reaction conditions and their corresponding drug loading capacities with average diameter. Condition No. 2 was chosen as optimal for synthesis of DOX-conjugated organosilica nanoparticles [46].

No.	Si-PBA [mM]	Dox [mM]	APTMS [mM]	NaOH [mM]	PEI [mgmL^−1^]	DLC [%]	Diameter [nm]
1	10	1	5	0.1	4	31.7	246 ± 11
2	10	1	10	0.1	4	22.4	229 ± 9
3	10	1	20	0.1	4	15.3	1352 ± 162
4	10	1	10	0.1	2	40.0	410 ± 12
5	10	1	10	0.1	8	14.0	290 ± 3
6	10	1	10	0	4	22.6	517 ± 33
7	10	1	10	1	4	17.1	519 ± 20

**Table 2:** Different nanocarrier-based systems used for doxorubicin delivery on the market or in clinical trials [66].

Delivery System	Name	Composition	Indication	Status	Refs.
Liposome	Doxil^®^/Caelyx^®^	Hydrogenated soy phosphatidylcholine/cholesterol/methyl-distearoyl phosphoethanolamine-polyethylene glycol 2000	AIDS-related Kaposi’s sarcoma; ovarian cancer; metastatic breast cancer; multiple myeloma	Approved	[67,68]
	Myocet^®^	Phosphatidylcholine/cholesterol	Metastatic breast cancer	Approved	[69,70]
	Lipo-Dox^®^	1,2-Distearoyl-sn-glycero-3-phosphocholine/polyethylene glycol	AIDS-related Kaposi’s sarcoma; ovarian cancer; metastatic breast cancer	Approved in Taiwan	[71,72]
	ThermoDox^®^	1,2-Dipalmitoyl-sn-glycero-3-phosphatidylcholine/1-stearoyl2-hydroxy-sn-glycero-3-phosphatidylcholine/1,2-distearoylsn-glycero-3-phosphoethanolamineN-methoxypoly-ethyleneglycol 2000	Primary liver cancer	Failed Phase III	[73]
	MM-302	PEG-modified liposomes containing doxorubicin and HER2-specific antibodies	Advanced breast cancer	Phase II	[3,74]
	2B3-101	Glutathione PEGylated liposomal doxorubicinhydrochloride	Breast cancer metastases in the brain	Phase II	[74]
Nanoparticle	Livatag^®^	Polyisohexylcyanoacrylate	Primary liver cancer	Failed Phase III	[3]
Polymer–drug conjugate	FCE28068/PK1	N-(2-Hydroxypropyl)methacrylamidedoxorubicin	Breast cancer; non-small cell lung cancer; colorectal cancer	Phase II	[66]
	FCE28069/PK2	Galactosamine-N-(2-hydroxypropyl)methacrylamidedoxorubicin	Primary or metastatic liver cancer	Phase II	[66]
Polymeric micelle	SP1049C	Pluronics^®^ L61/F127	Adenocarcinoma of esophagus and gastroesophageal junction	Phase III	[66]
	NK911	Doxorubicin-conjugated poly-aspartic acid/polyethylene glycol	Metastatic pancreatic cancer	Phase II	[66]

## Data Availability

No new data were created or analyzed in this study. Data sharing does not apply to this article.
